# Supplementary material for: Seizures in PPT1 Knock-In Mice Are Associated with Inflammatory Activation of Microglia
Source: Int J Mol Sci. 2022 May 17;23(10):5586. doi: 10.3390/ijms23105586 (PMC9144763; doi:10.3390/ijms23105586)
Supplement: Supplementary file 1 [file ijms-23-05586-s001.zip › ijms-1676112-supplementary.pdf]

**Supplementary materials**

## 1. Genotyping for identifying PPT1 KI and WT

The male and female PPT1 knock-in offspring with WT/c.451C>T mutation were used as breeders to generate enough reservoirs of PPT1 KI mice in the current study. These breeders carried out yielding WT, WT/c.451C>T, and c.451C>T/c.451C>T (PPT1 KI) mice. The genotypes of PPT1 KI and its wild-type littermate were identified by PCR. Figure S1 demonstrates the representative genotyping data of PPT1 KI and WT mice.

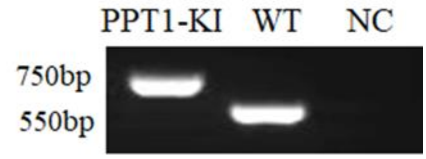


**Figure S1.** Representative blot of PPT1 KI mice genotyping. PPT1-KI, PPT1 c.451C>T/c.451C>T mouse; WT, wild-type littermate; NC, negative control.

## 2. Behavioral study

- (1) Clasping behavior in PPT1 KI mice

Hind-limb clasping during tail suspension has been used as an indicator of neurological dysfunction in various animal models of neurological disease (Lalonde and Strazielle, 2011). In the test, mice were suspended by the base of the tail and videotaped for 10–15 seconds. Three separate trials were performed for 3 consecutive days, and a representative picture was is in SF2. The clasping behavior was not manifested in WT mice but can be easily seen in their Ppt1-KI littermates over 6-month-old mice.

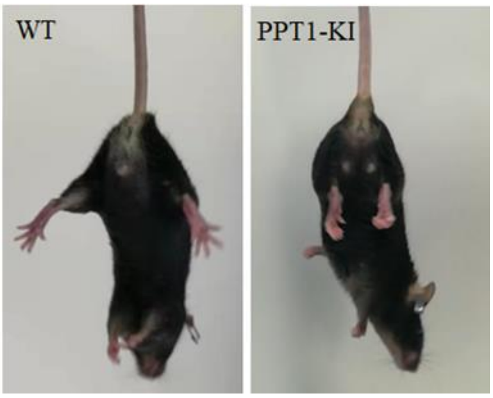


**Figure S2.** Representative pictures of clasping behavior in PPT1 KI mouse compared to the wild-type mouse. WT, wild-type littermate; PPT1 KI, PPT1 knock-in c.451C>T/c.451C>T mouse.

- (2) Videorecording of seizure activity in PPT1 KI and WT littermate

PPT1 KI mice aged 5–7 months and 7-month-old WT mice were placed into the observation cage with water and food at room temperature (25 ± 2 °C). Mice were videotaped by the Plexon CinePLex Studio Application Version 3.7 (Plexon, USA) for 24 h in 12 h/12 h day/night cycle (lights on at 8 a.m.). The video was edited by Adobe illustrator CS6.

Video clips for seizure recordings of PPT1 KI mice were uploaded to Google Drive, which can be shared at the link: https://drive.google.com/drive/my-drive.

### 3. Age-related changes in the expression of GFAP and GluN2B in PPT1 KI mice

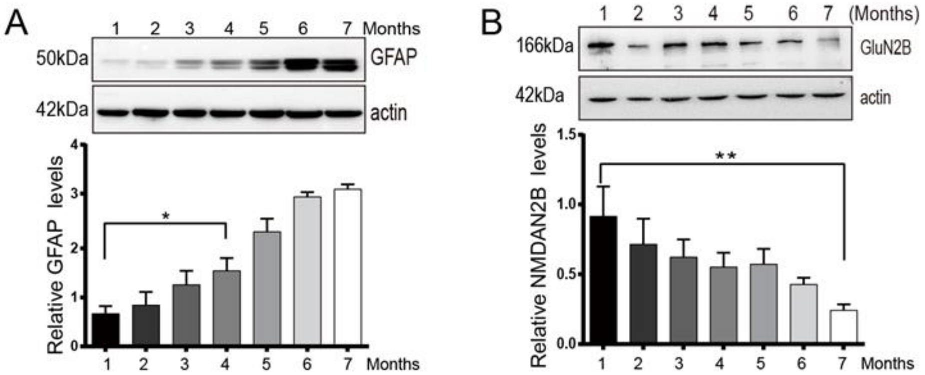


**Figure S3.** Representative Western blots (top) and bar graphs (bottom) showing expressions of GFAP and GluN2B in the hippocampus of PPT1 KI mice from 1 to 7 months old; (**A**) GFAP, (1 month old vs. 4 months old, $p = 0.01$, $n = 21$); (**B**) GluN2B, (1 month old vs. 7 months old, $p < 0.01$, $n = 35$).
